# Supplementary material for: Integration of Genomic and Clinical Retrospective Data to Predict Endometrioid Endometrial Cancer Recurrence
Source: Int J Mol Sci. 2022 Dec 16;23(24):16014. doi: 10.3390/ijms232416014 (PMC9785370; doi:10.3390/ijms232416014)
Supplement: Supplementary file 1 [file ijms-23-16014-s001.zip › ijms-2055540-supplementary.pdf]

International Journal of *Molecular Sciences*

MDPI

Article

Integration of Genomic and Clinical Retrospective Data to Predict Endometrioid Endometrial Cancer Recurrence

Jesus Gonzalez-Bosquet [1,*], Sofia Gabrilovich [1], Megan E. McDonald [1], Brian J. Smith [2], Kimberly K. Leslie [3], David D. Bender [1], Michael J. Goodheart [1] and Eric Devor [1]

[1] Department of Obstetrics and Gynecology, University of Iowa, 200 Hawkins dr., Iowa City, IA 52242, USA
[2] Department of Biostatistics, University of Iowa, 145 N Riverside Dr., Iowa City, IA 52242, USA
[3] Division of Molecular Medicine, Departments of Internal Medicine and Obstetrics and Gynecology, The University of New Mexico Comprehensive Cancer Center, 915 Camino de Salud, CRF 117, Albuquerque, NM 87131, USA
* Correspondence: jesus-gonzalezbosquet@uiowa.edu; Tel.: +1-(319)-356-2160; Fax: +1-(319)-353-8363

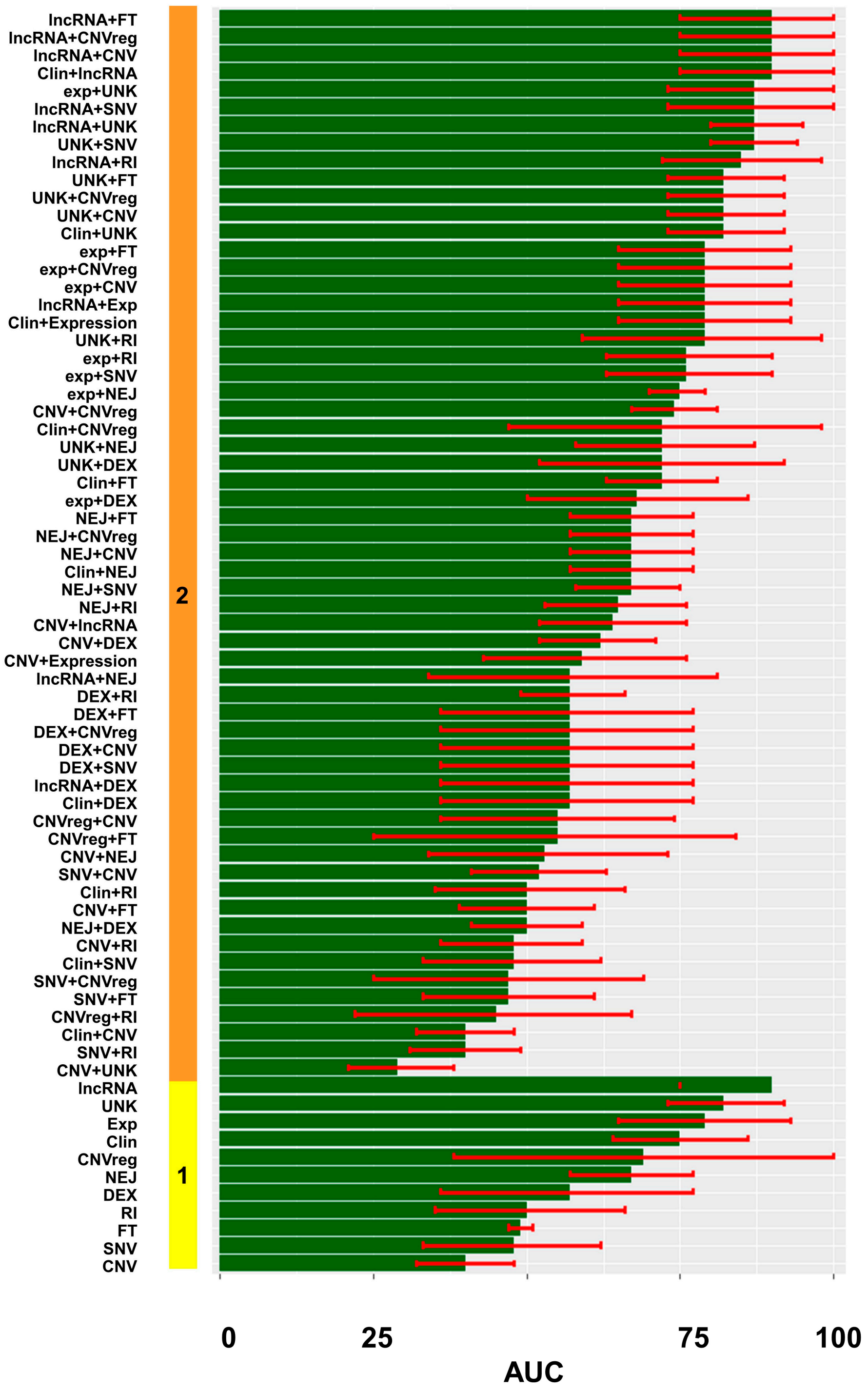

Supplementary Figure S1: Performance of prediction models of endometrial cancer recurrence for 1 and 2 types of variables.

The solid vertical bar represents the number of types of data: 1 (yellow): only one variable was included in the model; 2 (orange): combination of 2 types of variables.

Different performances on both panels are displayed in ascending order. The x axis is AUC as a percentage (0-100%). The red error mark displays the 95% confidence interval (CI). Overall, 71 models with different combinations of data were tested. *Transcriptome*: Exp: gene expression; DEX: exon expression; lncRNA: long non-coding RNA. *Genomic variation*: SNV: single nucleotide variation; CNV: gene copy number by gene; CNVreg: copy number by chromosomal region. *Structural variation*: FT: Fusion transcripts; RI: Retained intron; NEJ: Novel exon/junction; UNK: Unknown SV. Graphics were generated with R package *ggplot*.

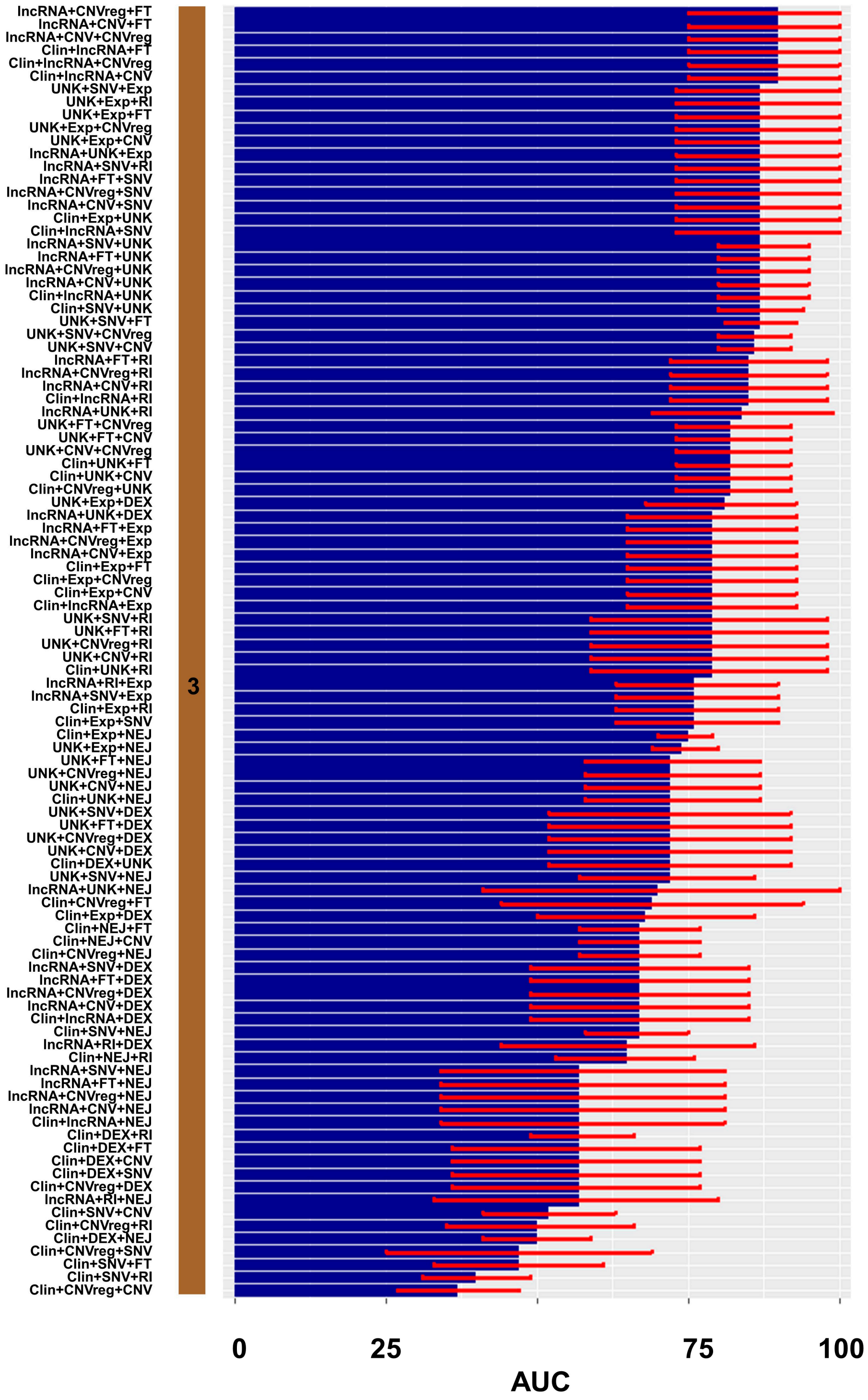

Supplementary Figure S2: Performance of prediction models of endometrial cancer recurrence for 3 types of variables.

These are the 100 prediction models with combination of 3 types of variables (left marron bar). As before, different performances on both panels are displayed in ascending order. The x axis is AUC as a percentage (0-100%). The red error mark displays the 95% confidence interval (CI). Overall, 100 models with different combinations of data were tested. *Transcriptome*: Exp: gene expression; DEX: exon expression; lncRNA: long non-coding RNA. *Genomic variation*: SNV: single nucleotide variation; CNV: gene copy number by gene; CNVreg: copy number by chromosomal region. *Structural variation*: FT: Fusion transcripts; RI: Retained intron; NEJ: Novel exon/junction; UNK: Unknown SV. Graphics were generated with R package *ggplot*.

		Recurrent (N=60)	Non-recurrent (N=346)	p-value
**Age**	(average)	63	62	0.555
**BMI**	(average)	32.8	33.2	0.853
**Grade**				0.003*
	1	5	92	
	2	18	99	
	3	137	155	
**MI**				0.282
	<50%	28	237	
	>50%	4	18	
	unknown	28	91	
**Cytology**				0.060
	No	40	234	
	Yes	8	20	
**Stage**				<0.001
	I	32	255	
	II	41	31	
	III	16	54	
	IV	8	6	

Supplementary Figure S3: TCGA clinical patient characteristics. These are the baseline variables determined at treatment completion and included in the analysis. MI: myometrial invasion (present in <50% and >50%). In TCGA dataset there are similar number or recurrences than in the study population from the UI (p-value=0.59)

*Statistically significant with P-value < 0.05.

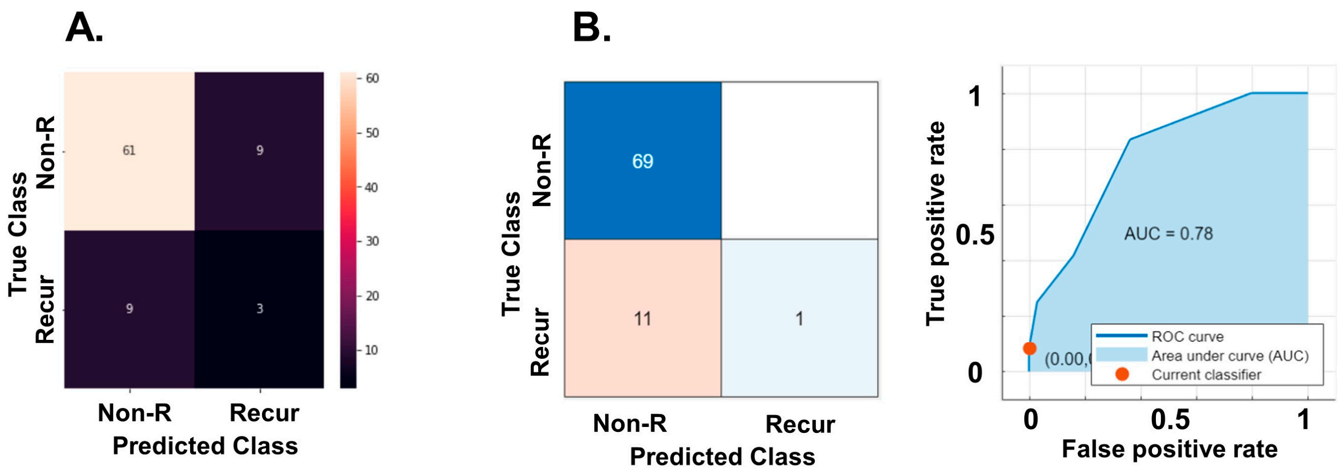

Supplementary Figure S4: Validation of the best prediction model in an independent dataset (TCGA).
A. Validation of the best model only lncRNA data and no clinical variables. These are the results after the training and validating the model with TensorFlow in 75% of the samples, and then performing testing in the resting 25% of data. We are showing the confusion matrix of the testing results: with the true (True Class) versus the predicted values (Predicted Class). AUC of 0.68 and accuracy of 0.78. Recur: Recurrence; Non-R: non recurrent.
B. Validation of the best model with only lncRNA data and no clinical variables with MATLAB platform. We are showing testing results in 20% of the data, after training and validating have been performed. In 15 of them the accuracy of testing was over 85%%. Specifically, this is the cosine KNN (K-Nearest Neighbors) method. The left panel shows the confusion matrix representing the true (True Class) versus the predicted values (Predicted Class). The right panel is an ROC graphic: true positives in the x axis, false positives in the y axis, and AUC results. AUC of 0.78 and accuracy of 86%.

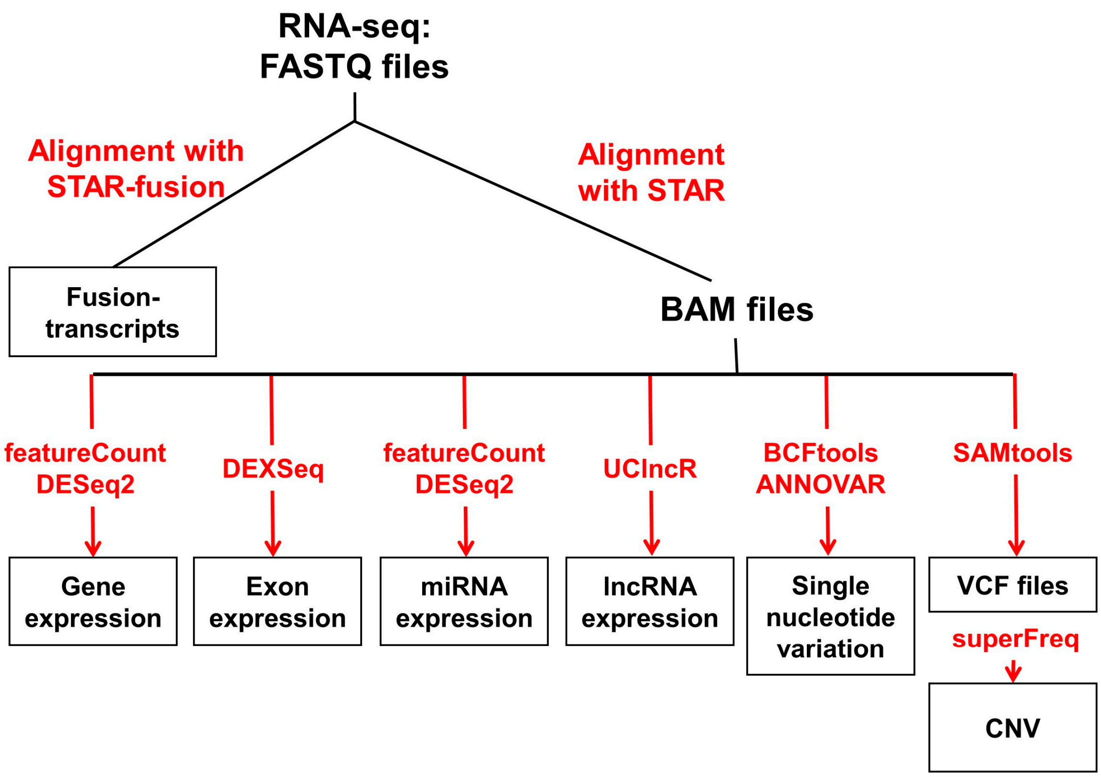

Supplementary Figure S5: Pipeline of genomic analytics starting with RNA sequencing.
From fastq files originated from RNA-seq, we created fusion transcripts and BAM files. The rest of genomic elements were produced from these BAM files and different software analytics. In red, different software utilities used to generate genomic elements for this project.
